# Synthesis and Properties of the Novel High-Performance Hydroxyl-Terminated Liquid Fluoroelastomer

**DOI:** 10.3390/polym15112574

**Published:** 2023-06-04

**Authors:** Donghan Li, Chen Yang, Ping Li, Lu Yu, Shufa Zhao, Long Li, Hailan Kang, Feng Yang, Qinghong Fang

**Affiliations:** 1 College of Materials Science and Engineering, Shenyang University of Chemical Technology, Shenyang 110142, China; 2 Liaoning Provincial Key Laboratory of Rubber & Elastomer, Shenyang University of Chemical Technology, Shenyang 110142, China; 3 Shenyang Guide Rubber Products Co., Ltd., Shenyang 110141, China

**Keywords:** liquid fluoroelastomer, LiAlH_4_, reduction, hydroxyl

## Abstract

Functional liquid fluoroelastomers are in high demand in new energy fields. And these materials have potential applications in high-performance sealing materials and as electrode materials. In this study, a novel high-performance hydroxyl-terminated liquid fluoroelastomer (t-HTLF) with a high fluorine content, temperature resistance, and curing efficiency was synthesised from a terpolymer of vinylidene fluoride (VDF), tetrafluoroethylene (TFE), and hexafluoropylene (HFP). A carboxyl-terminated liquid fluoroelastomer (t-CTLF) with controllable molar mass and end-group content was first prepared from a poly(VDF-*ter*-TFE-*ter*-HFP) terpolymer using a unique oxidative degradation method. Subsequently, an efficient “one-step” reduction of the carboxyl groups (COOH) in t-CTLF into hydroxyl groups (OH) was achieved via the functional-group conversion method using lithium aluminium hydride (LiAlH_4_) as the reductant. Thus, t-HTLF with a controllable molar mass and end-group content and highly active end groups was synthesised. Owing to the efficient curing reaction between OH and isocyanate groups (NCO), the cured t-HTLF exhibits good surface properties, thermal properties, and chemical stability. The thermal decomposition temperature (T_d_) of the cured t-HTLF reaches 334 °C, and it exhibits hydrophobicity. The oxidative degradation, reduction, and curing reaction mechanisms were also determined. The effects of solvent dosage, reaction temperature, reaction time, and ratio of the reductant to the COOH content on the carboxyl conversion were also systematically investigated. An efficient reduction system comprising LiAlH_4_ can not only achieve an efficient conversion of the COOH groups in t-CTLF to OH groups but also the in situ hydrogenation and addition reactions of residual double bonds (C=C) groups in the chain, such that the thermal stability and terminal activity of the product are improved while maintaining a high fluorine content.

## 1. Introduction

Fluoroelastomers are synthetic polymer elastomers containing fluorine atoms on the carbon atoms of their main or side chains [1]. At present, the commonly used fluoroelastomers are poly(VDF-*co*-CTFE) copolymer, poly(VDF-*co*-HFP) copolymer, and poly(VDF-*ter*-TFE-*ter*-HFP) terpolymer [2,3]. There are also some fluoroelastomers with special properties, mainly fluorosilicone rubber and fluorinated phosphazene rubber. Fluorosilicone rubber is an elastomer with trifluoropropyl side chain in the main chain of methyl vinyl siloxane, which is mainly used in aerospace, automobile, chemical industry and other fields. However, the fluorine content of fluorosilicone rubber is lower than that of fluoroelastomer, so its heat resistance and medium resistance are lower than that of fluoroelastomers, which limits its application fields [4]. Fluorinated phosphazene rubber is an elastomer whose molecular main chain consists of phosphorus atoms and nitrogen atoms alternately, and the phosphorus atoms have fluorinated alkoxy groups. It has excellent oxidation resistance, thermal stability and flame retardancy, but its application range is limited by the high production and other comprehensive costs [5]. Therefore, the focus of this paper is fluoroelastomers, especially terpolymers. The thermal stabilities and mechanical properties of terpolymers are better than those of copolymers [6,7]. The poly(VDF-*ter*-TFE-*ter*-HFP) terpolymer, a terpolymer of VDF, TFE, and HFP, is a high-quality material used in aviation, aerospace, automotive, and other industries [8]. It has a higher fluorine content than poly(VDF-*co*-HFP) copolymers and exhibits good heat and solvent resistances [9,10]. However, solid fluoroelastomers have disadvantages, such as poor processing properties, difficult forming and processing, and complicated vulcanisation methods. Therefore, processing and shaping these fluoroelastomers is difficult, and they cannot fully meet the demands of the transportation, chemicals, petroleum, and defence industries. Therefore, attention is turned to other liquid elastomers. However, the thermal stabilities and oxidation and chemical resistances of other liquid elastomers cannot be compared with those of solid fluoroelastomers. Therefore, liquid fluoroelastomers, which are low-molecular-weight fluoropolymers with good fluidities and plasticities, were developed.

Liquid fluoroelastomers are high fluorine-containing oligomers with active functional groups at both ends of the molecular chain [11]. After the end groups are converted into target functional groups, they exhibit conductivity and anti-fouling, self-repair, high strength, and high-speed curing properties [12,13,14,15]. They can be used as prepolymers or precursors of new functional polymer materials [16,17]. Liquid fluoroelastomers can be prepared using polymerisation and oxidative degradation methods [18]. Polymerisation methods include Iodine transfer copolymerization and functional group initiation, and oxidative degradation methods include one- and two-step oxidative degradation method.

Based on the synthetic process, liquid fluoroelastomers were first developed and industrially produced by DuPont Co. (Wilmington, DE, USA) and 3M Co. (Saint Paul, MN, USA) in the 1960s, with Japan and Russia catching up later. Viton, a representative liquid fluoroelastomer marketed by DuPont Co. (USA), can withstand low temperatures of up to −40 °C and does not require a separate agent for vulcanisation. A SIFEL-type liquid fluoroelastomer marketed by Shin-Etsu Co. (Tokyo, Japan) and Daikin Co. (Osaka, Japan) can be processed at 70–150 °C and has excellent solvent and low-temperature resistance, while the liquid fluoroelastomers developed by the Lebedev Synthetic Rubber Research Institute in Russia (CKΦ-26 and 120X) exhibit strong environmental resistance, withstand low temperatures of up to −35 °C, and can be processed at 20–70 °C [19,20]. Among them, liquid fluoroelastomer with excellent properties, controllable molar mass and narrow molar mass distribution can be synthesized by Iodine transfer copolymerization, so it is booming. The reaction system includes iodide, free radical initiator and comonomer. The comonomer is VDF and other fluorine-containing olefins, such as TFE, HFP and perfluorocyclobutene, and the iodide can be 1,4-diiodoperfluorobutane. The reaction conforms to the mechanism of free radical polymerization. In polymerization, α, ω-perfluoroalkyl diiodide is often used to synthesize fluoroelastomer precursors, and then other monomers such as VDF are initiated to polymerize [21]. Daiel brand liquid fluoroelastomer was produced by polymerization of α, ω-perfluoroalkyl diiodide with VDF, HFP and TFE in a certain proportion in Daikin Company of Japan. The product not only has excellent thermal stability (T_d_ is 380–400 °C), good chemical resistance, weather resistance and excellent surface properties, but also the mechanical properties of the cured product are similar to those of solid fluoroelastomer. DuPont Co. (Wilmington, USA) also synthesized liquid fluoroelastomer by a similar method [22]. Kostovet [23] provided some strategies to synthesize photo-cross-linkable telechelic diacrylate poly(VDF-*co*-PMVE) co-oligomersby changing iodines into “acrylate groups”. When using acryloyl chlorides as acrylating agents, the transformationwas most successful, and the product has good low temperature resistance. On this basis, liquid fluoroelastomer with end groups of hydroxyl, vinyl and carboxyl can be efficiently synthesized by hydrolysis, esterification, reduction and other methods, which has practical value in the fields of transportation and aerospace. In recent years, the oxidative degradation method has attracted extensive attention for the oxidative degradation of poly(VDF-*co*-HFP) copolymers because it is simpler and more efficient for preparing liquid fluoroelastomers than polymerisation. Li [24] used a poly(VDF-*co*-HFP) copolymer as a raw material to prepare carboxyl-terminated liquid fluoroelastomer (c-CTLF) via oxidative degradation method. The effects of various solvents and bases on the molar masses of the products, yield, and end-group conversion were systematically investigated. With the use of acetone as the solvent and potassium hydroxide (KOH) as the alkali, the molar mass of the product was 2.2 × 10^3^ g/mol, the yield was up to 98%, and the carboxyl content was up to 2.95 wt%. However, the oxidative degradation mechanism still needs to be thoroughly studied. Li [25] elucidated the primary and secondary relationships of the rules of the elimination reaction for poly(VDF-*co*-HFP) copolymers under alkaline conditions, the sequence structure and content of C=C, and a detailed reaction mechanism. The poly(VDF-*co*-HFP) copolymer undergoes the dehydrofluorination reaction mainly via the Zaitsev rule and via the Hofmann rule to a lesser extent. However, because of the complex molecular chain structures of the terpolymers, reports on terpolymer research are limited.

Recently, Li [26] discovered, after an extensive investigation, that the C=C in a chain significantly affects its thermal stability. On this basis, the oxidative degradation of the poly(VDF-*ter*-TFE-*ter*-HFP) terpolymer was proposed, and a novel and efficient t-CTLF was prepared. Although t-CTLF can be prepared via the oxidative degradation method with a controlled molar mass and functional-group content [27,28], the nature of the end carboxyl groups determines the defects, such as high curing temperature and poor thermal stability, of the cured products, which affect their overall performance and shorten their service lives. Currently, liquid fluoroelastomers with high fluorine contents, high-temperature resistances, and highly active end groups are being developed. The terminal hydroxyl group exhibits better surface and interface properties and is compatible with metals and inorganic substances. Therefore, the conversion of COOH to OH is of interest [29,30] and is commonly achieved using sodium borohydride (NaBH_4_) systems [31].

Although NaBH_4_ is frequently employed as a reductant [32,33], it typically cannot directly reduce COOH to OH [34] and is commonly used in conjunction with iodine or metal salts. Studies on the efficient conversion of COOH to OH using complicated NaBH_4_ systems have recently been reported. By reducing COOH to OH using a NaBH_4_/I_2_ reduction system to prepare c-CTLF via oxidative degradation method, Wu and Li [35,36] synthesised a copolymer called hydroxyl-terminated liquid fluoroelastomer (c-HTLF) with a hydroxyl content of 1.0–2.30 wt%. Reaction conversion of 88–100% could be achieved by carefully controlling the reaction conditions. Li [37] conducted a preliminary investigation on the use of a sodium borohydride/rare earth chloride (NaBH_4_/RECl_3_) system in liquid fluoroelastomers using c-CTLF as the raw material and three reduction systems (NaBH_4_/CeCl_3_, NaBH_4_/LaCl_3_, and NaBH_4_/NdCl_3_). The highest COOH conversion was achieved when NaBH_4_/CeCl_3_ was used. On this basis, Chang [38] synthesised c-HTLF using a one-pot method, with NaBH_4_/SmCl_3_ as the reduction system. COOH was effectively reduced with 92% conversion under the optimal reaction conditions. Chang [39] studied the reduction of c-CTLF by sodium borohydride using a metal chloride (NaBH_4_/MCl_x_) system and the reduction mechanism. The C=C and COOH groups of c-CTLF could be eliminated, with the rare-earth metal MCl_x_ reduction system exhibiting a more significant reduction effect than the transition-metal MCl_x_ reduction system. Because the LiAlH_4_ reaction system does not require the addition of a co-catalyst and can complete the reduction with high efficiency in a single step, it is typically preferred over the NaBH_4_ composite system to reduce c-HTLF for meeting the requirement for this material in the new energy field.

LiAlH_4_ is a stronger reductant than NaBH_4,_ and it can attribute some spatial selectivity to the reaction [40,41]. Using LiAlH_4_ as the reductant, Duan [42] reduced COOH to OH in c-CTLF at a carboxyl concentration as low as 0.12 wt% with a conversion rate as high as 95%. Wen [43] reduced c-CTLF to c-HTLF using two reduction systems: diisobutylaluminum hydride (DIBAl-H)/LiAlH_4_ and triisobutylaluminum [Al(i-Bu)_3_]/LiAlH_4_. The reducing abilities of the two systems were also evaluated. DIBAl-H/LiAlH_4_ was better suited for reducing c-CTLF at 60 °C. The experimental procedure was straightforward when LiAlH_4_ was used as the reductant, and the reaction was safer. Additionally, because of the high hydrogen content of the LiAlH_4_ molecule, a smaller amount of the reagent is required to provide a greater reduction effect, which significantly reduces manufacturing costs. Consequently, LiAlH_4_ has many applications in fine chemicals, medicinal synthesis, and energy production [44,45,46,47,48].

Few reports exist on the preparation methods, reaction mechanisms, structures, properties, and curing of t-HTLF. Thus, to design and synthesise t-HTLF and elucidate the relationship between its molecular chain structure and properties, we used the oxidative degradation and functional-group conversion methods to prepare t-CTLF and t-HTLF, respectively. The poly(VDF-*ter*-TFE-*ter*-HFP) terpolymer was selected as the raw material, and fluoroelastomer formation was confirmed via spectral analyses and chemical quantification. Furthermore, property investigations and comparative analyses of the relationship between the structure and properties of the cured t-HTLF were performed.

## 2. Materials and Methods

### 2.1. Experimental Materials

Poly(VDF-*ter*-TFE-*ter*-HFP) terpolymer was purchased from Chenguang Research Institute of Chemical Industry (Zigong, China). Acetone (Analytical Reagent (AR)), KOH, (AR), a hydrogen peroxide solution with a mass fraction of 30% (H_2_O_2_, AR), concentrated hydrochloric acid (HCl, AR), benzyl triethyl ammonium chloride (BTEAC), cyclohexane, 3# Jet aircraft oil and concentrated sulfuric acid (H_2_SO_4_, AR) were purchased from Shanghai Aladdin Biochemical Technology Co., Ltd. (Shanghai, China). LiAlH_4_ (AR), anhydrous ethanol (C_2_H_5_OH) and tetrahydrofuran (THF, AR) were purchased from Tianjin Comio Chemical Reagent Co., Ltd. (Tianjin, China).

### 2.2. Experimental Apparatus

#### 2.2.1. Spectral Analyses

Attenuated total reflectance/Fourier transform infrared (ATR-FTIR) spectroscopy was performed using a Thermo Fisher Scientific Nicolet is10 infrared spectrometer with a scan range of 600–4000 cm^−1^ over 16 scans. 

^1^H nuclear mangnetic resonance (^1^H-NMR) was performed using an AVANCE-III-500 MHz spectrometer (Bruker, Switzerland) using deuterated acetone (C_3_D_6_O) as the solvent and tetramethylsilane (TMS) as the standard.

^19^F-NMR was performed using an AVANCE-NEO-400 MHz spectrometer (Bruker, Switzerland), and the standard was monofluorotrichloromethane (CFCl_3_).

Solid-state ^19^F-NMR was performed using an JEOL JNM ECZ600R spectrometer (Japan), and the specific conditions were frequency of 564 MHz, pulse width of 90 deg and rotating speed of 21 kHz.

#### 2.2.2. Determination of Molar Mass

The molar mass (M_n_) and polydispersity index (PD) of the fluoroelastomers was estimated by gel permeation chromatography (GPC) system (PL-GPC50) from Varian, Inc. Company. Polystyrene (PS) was used as the standard sample and chromatographic grade tetrahydrofuran (HPLC) as the mobile phase at a flow rate of 1 mL/min and a test temperature of 30 °C.

#### 2.2.3. Thermal Properties

Differential scanning calorimetry (DSC) measurements were performed using a TA thermal analyser (Newcastle, USA). The test conditions were as follows: nitrogen atmosphere; constant temperature of 40 °C; first ramp up, then ramp down and then ramp up; temperature range of 10–100 °C; and ramp-up rate of 10 °C/min to test the glass transition temperature (T_g_) of the products.

The thermogravimetric analysis (TGA) measurements were performed using a TA Instruments-Waters LLC thermal weight-loss analyser with the following test conditions: T_d_ of the product was tested under a nitrogen atmosphere in a temperature range of 30–600 °C with a ramp-up rate of 10 °C/min.

#### 2.2.4. Mechanical Properties

The mechanical properties of the samples were evaluated using the GB/T528-2009 standard and an Instron 3365 universal tensile-testing machine. At least 5 samples were tested at a tensile rate of 500 mm/min at a test temperature of 25 °C. The average value was then calculated.

#### 2.2.5. Surface Properties

The static contact-angle test was performed using a German Dataphysics OCA20 instrument with a test water volume of 5 μL. Points at 5 locations on the sample to be tested were selected and tested, and the average was considered the water contact angle (WCA) of the sample.

#### 2.2.6. Oil and Acid Resistances

The swelling degree of cured t-HTLF was tested according to the method described in GB/T 1690-2006 using 3# jet aircraft oil, cyclohexane, 36.50 wt% HCl, and 50.00 wt% H_2_SO_4_ as solvents. The cured product (2.00 g) was soaked in the desired solvent for 24.0 or 72.0 h at 25 ± 2 °C, removed, dried with filter paper, and weighed. The swelling degree w% is calculated by Equation (1):

(1) $$w\%=\frac{m_{2}-m_{1}}{m_{1}}\times 100\%$$

In the Equation (1), m_1_ is the mass of the sample before soaking, and m_2_ is the mass of the sample after soaking, both in g.

### 2.3. Quantitative Analysis via Chemical Titration

#### 2.3.1. Determination of the Carboxyl Content

In 40 mL of acetone, 1 g of t-CTLF was dissolved. To this solution, 0.1 mL of bromothymol blue indicator was added at a concentration of 10 g/L, and the solution was titrated with a KOH/C_2_H_5_OH (0.1 mol/L) solution until it turned green, which was the endpoint of the titration. The carboxyl content was calculated using the following Equation (2):

(2) $$COOH\%=\frac{V\times C\times 45.02}{m}\times 100\%$$

where V is the volume of the KOH/C_2_H_5_OH solution consumed for titration (mL), C is the concentration of the KOH/C_2_H_5_OH standard titration solution (mol/L), and m is the mass of the test sample (mg).

#### 2.3.2. Determination of the Carboxyl Conversion Rate

The carboxyl contents in t-CTLF and t-HTLF were determined via chemical titration; the difference between them was the hydroxyl content, which was calculated using the following Equation (3):

(3) $$OH\%=w_{0}-w_{1}$$

Further calculations yielded the carboxyl conversion rate using the following Equation (4):

(4) $$\alpha \%=\frac{w_{0}-w_{1}}{w_{0}}\times 100\%$$

In the equations, w_0_ represents the carboxyl content in t-CTLF, and w_1_ represents the carboxyl content in t-HTLF.

### 2.4. Sample Preparation

#### 2.4.1. Carboxyl-Terminated Liquid Fluoroelastomer

Poly(VDF-*ter*-TFE-*ter*-HFP) terpolymer (20 g) and acetone (500 mL) were added to a 1000 mL flask. The mixture was stirred at room temperature until the terpolymer had dissolved completely. Under stirring at 0 °C, BTEAC, KOH and 30 wt% H_2_O_2_ aqueous solutions were added sequentially, and stirred for 7 h. After the reaction, HCl was used to acidify the mixture, and excess deionised water was added. The resultant product was concentrated using a rotary evaporator and dried at 65 °C under vacuum until constant weight, and a light yellow viscous liquid was obtained.

#### 2.4.2. Hydroxyl-Terminated Liquid Fluoroelastomer

The t-CTLF (10 g, COOH content 3.24 wt%) and THF (100 mL) were added to a 250 mL flask. Until the liquid fluoroelastomer had dissolved completely, LiAlH_4_ was slowly added to the mixture at room temperature. The temperature was then increased to 80 °C, and the reaction was allowed to proceed for 4 h. Subsequently, LiAlH_4_ was neutralised with HCl, and the mixture was filtered. The product was collected until constant weight after drying at 65 °C under vacuum to obtain a yellow viscous liquid.

#### 2.4.3. Curing

The t-HTLF (10 g, OH content 3.05 wt%) and acetone (10 mL) were added to a beaker, according to the OH/NCO molar ratio of 1.00/1.20 weighed HDI trimer, and then dissolved in acetone. The two were uniformly mixed and dried in an oven at 60 °C to remove the solvent. At a solvent content of 3–5 mL, the solution was injected into a preheated mould at 60 °C, which was then placed in a vacuum drying oven at 60 °C and left there for 8–48 h to remove the solvent. Subsequently, the temperature was increased to 90 °C, which was the curing temperature, for 4 h to obtain a light-yellow curing product.

**Scheme 1:**
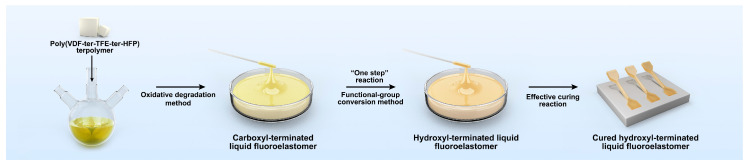
The synthesis and curing route of hydroxyl-terminated liquid fluoroelastomer.

The synthesis and curing route of hydroxyl-terminated liquid fluoroelastomer is shown in Scheme 1.

## 3. Results and Discussion

### 3.1. Structural Characterisation of the Liquid Fluoroelastomer

#### 3.1.1. FTIR Spectra of the Liquid Fluoroelastomers

**Figure 1:**
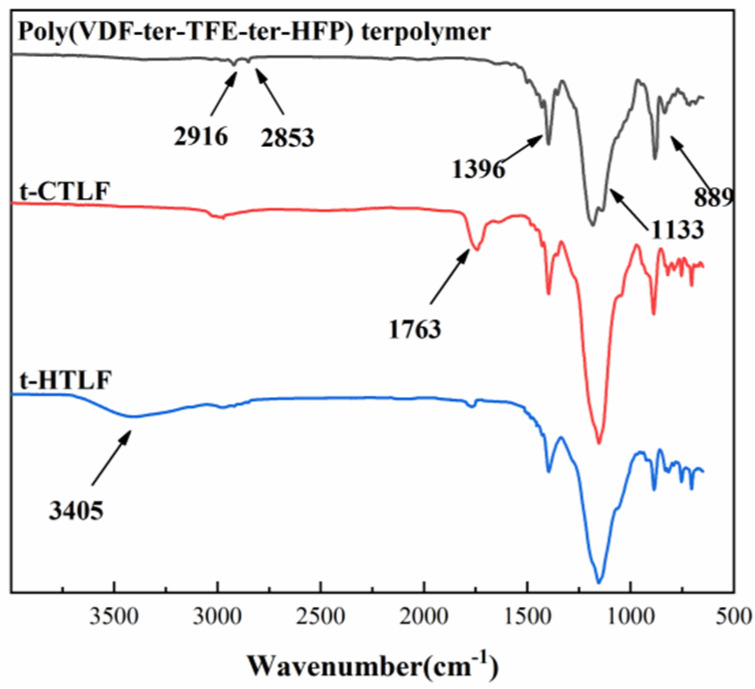
FTIR spectra of the poly(VDF-*ter*-TFE-*ter*-HFP) terpolymer, t-CTLF, and t-HTLF.

As shown in Figure 1, the FTIR spectra exhibit absorption peaks at 870–890 cm^−1^, 1155–1180 cm^−1^, and 1395–1400 cm^−1^, which may be ascribed to the stretching vibrations of -CF-, -CF_2_-, and -CF_3_-, respectively [49]. A comparison of the FTIR spectra of t-CTLF and t-HTLF after the reduction reaction indicate that the peaks at approximately 1763 cm^−1^, which is assigned to the COOH, decrease significantly, while a new peak appears at 3405 cm^−1^, which is assigned to the OH. Thus, t-HTLF had been successfully synthesised [50].

#### 3.1.2. ^1^H-NMR Spectra of the Liquid Fluoroelastomers

**Figure 2:**
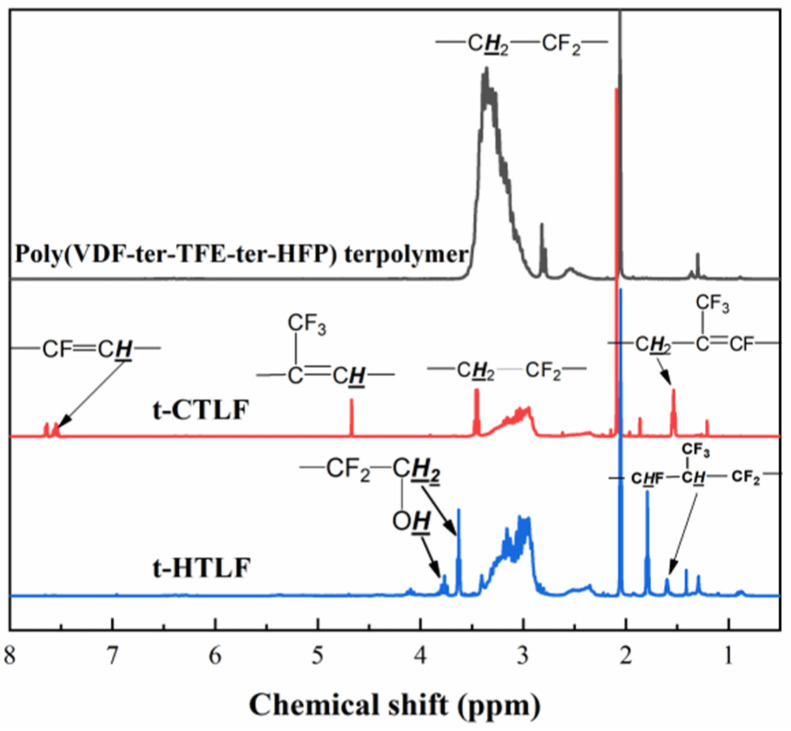
^1^H-NMR spectra of the poly(VDF-*ter*-TFE-*ter*-HFP) terpolymer, t-CTLF, and t-HTLF.

Figure 2 shows the characteristic peak corresponding to the -CH_2_CF_2_- structure at 3.51–2.30 ppm for the three curves. The t-CTLF also exhibits distinct peaks corresponding to the C=C structure at δ = 1.55 and 4.68 ppm. At 4.68 ppm, the characteristic structural peaks of C=C disappear in the ^1^H-NMR spectra of the t-HTLF after the reaction. The characteristic structural peaks of -CH_2_OH also appear at δ = 3.63 and 3.73 ppm. This indicated that COOH in the t-CTLF was converted to OH and that C=C underwent hydrogenation and addition reaction. Thus, the LiAlH_4_ reduction system is effective and versatile.

#### 3.1.3. ^19^F-NMR Spectra of the Liquid Fluoroelastomers

**Figure 3:**
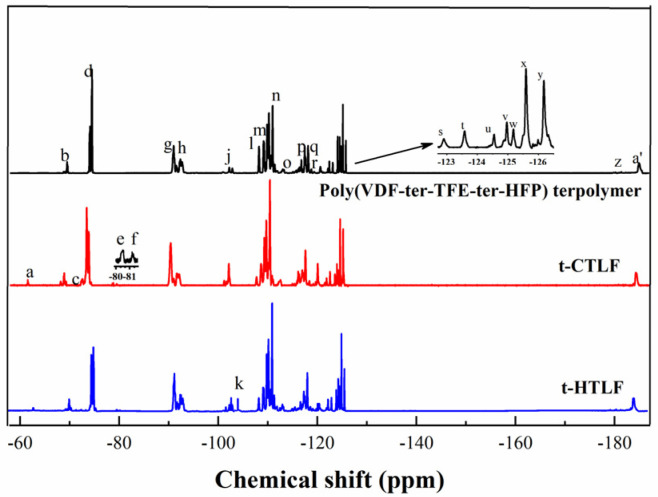
^19^F-NMR spectra of the poly(VDF-*ter*-TFE-*ter*-HFP) terpolymer, t-CTLF, and t-HTLF.

As seen in Figure 3, compared with the poly(VDF-*ter*-TFE-*ter*-HFP) terpolymer, t-CTLF shows a more intense structural characteristic peak corresponding to -CF_2_CF_2_COOH at δ = −63.53 ppm. However, the structural characteristic peak corresponding to -CF_2_CF_2_COOH in t-HTLF shows a significant weakening at δ = −63.53 ppm, while the characteristic peak corresponding to the -CF_2_CH_2_OH structure appears at δ = −104.02 ppm [51]. This indicates that the t-CTLF had been successfully reduced to t-HTLF, which is consistent with the ^1^H-NMR results.

The fluorine contents in the raw material and product molecular chains were further calculated [52], and the specific characteristic peaks are shown in Table 1. The characteristic peak area corresponding to the VDF sequence structure was set as ∑CF_2_ = I_−63.53_ + I_−91.95_ + I_−93.28_ + I_−95.64_ + I_−103.48_ + I_−109.85_ + I_−111.64_ + I_−113.85_ + I_−117.26_ + I_−118.68_. The characteristic peak area corresponding to the HFP sequence structure was set as ∑CF_3_ = I_−70.76_ + I_−75.44_ + 2(I_−74.29_ + I_−80.53_ + I_−81.19_). The characteristic peak area corresponding to the TFE sequence structure was set as ∑CF_2_ = I_−121.13_+ I_−122.90_+ I_−123.58_+ I_−124.54_+ I_−124.97_+ I_−125.19_+ I_−125.59_+ I_−126.18_. The monomer contents (X) of t-CTLF before and after the reduction reaction were calculated using the following Equations (5)–(7).

(5) $$X_{VDF}=\frac{\sum_{VDF}{CF}_{2}}{2/3\sum_{HFP}{CF}_{3}+\sum_{VDF}{CF}_{2}+\sum_{TFE}{CF}_{2}}\times 100\%$$

(6) $$X_{HFP}=\frac{2/3\sum_{HFP}CF_{3}}{2/3\sum_{HFP}CF_{3}+\sum_{VDF}{CF}_{2}+\sum_{TFE}{CF}_{2}}\times 100\%$$

(7) $$X_{TFE}=\frac{\sum_{TFE}{CF}_{2}}{2/3\sum_{HFP}CF_{3}+\sum_{VDF}{CF}_{2}+\sum_{TFE}{CF}_{2}}\times 100\%$$

The calculation yielded X_VDF_, X_HFP_ and X_TFE_, and the fluorine content (X_F_) in the sample was calculated according to the following Equation (8):

(8) $$X_{F}=\frac{X_{VDF}\times 38+X_{TFE}\times 76+X_{HFP}\times 114}{X_{VDF}\times 64+X_{TFE}\times 100+X_{HFP}\times 150}\times 100\%$$

According to the Equations (5)–(8), it can be calculated that the contents of VDF, HFP and TFE in t-CTLF were 66%, 17%, 17%, respectively; The contents of VDF, HFP and TFE in t-HTLF were 67%, 15% and 18%, respectively. After the reduction reaction, the fluorine content in the t-HTLF decreased from 68% to 67% of the raw material.

#### 3.1.4. GPC of the Liquid Fluoroelastomers

**Figure 4:**
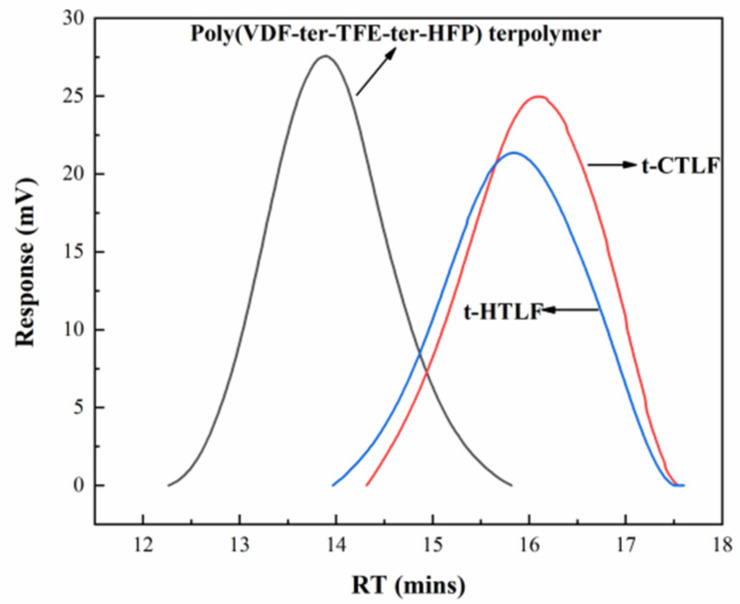
GPC spectra of the poly(VDF-*ter*-TFE-*ter*-HFP) terpolymer, t-CTLF, and t-HTLF.

As shown in Figure 4, the M_n_ of the t-CTLF decreased from 93.0 × 10^3^ g/mol to 3.8 × 10^3^ g/mol after oxidative degradation reaction. However, when COOH was converted to OH, the M_n_ of the t-HTLF increased to 4.2 × 10^3^ g/mol owing to the hydrogenation and addition of C=C on the molecular chain. The detailed values were shown in Table 2.

#### 3.1.5. Reaction Mechanisms

**Figure 5:**
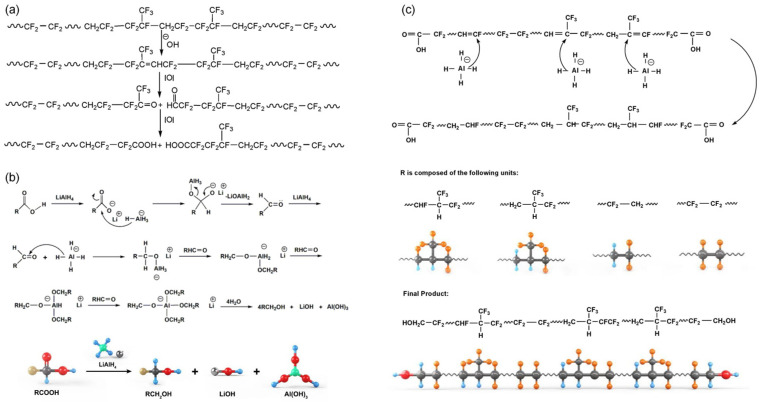
Reaction mechanism of the oxidative degradation reaction (**a**), the reduction reaction (**b**) and double bond hydrogenation addition (**c**).

The abovementioned results suggest that under the alkaline conditions of oxidative degradation, poly(VDF-*ter*-TFE-*ter*-HFP) is deprotonated at -CF_2_CH_2_- units, and the subsequent fluoride ion expulsion affords C=C bonds. These bonds are further oxidatively cleaved to afford t-CTLF (Figure 5a) [42]. Upon the further treatment with LiAlH_4_, the COOH groups of t-CTLF are converted to the corresponding Li salts, which are attacked by AlH_4_^−^ at the carbonyl carbon. The resulting unstable intermediate expels LiOAlH_3_^−^ to form an aldehyde, which reacts with another equivalent of LiAlH_4_ to yield an alcoholate of the RCH_2_OAlH_3_^−^ type and eventually afford the corresponding alcohol after hydrolysis (Figure 5b) [53,54,55]. Notably, all four hydride ions of AlH_4_^−^ can participate in reduction.

The efficient reduction system of LiAlH_4_ reported in this study can not only achieve an efficient conversion of COOH in the t-CTLF to OH but also the in situ hydrogenation addition reaction of the residual C=C in the chain. Thus, the t-HTLF not only has a high fluorine content but also an improved activity of its end groups. A “one step” reaction is thus achieved, along with several beneficial effects.

### 3.2. Thermal Properties of the Liquid Fluoroelastomers

**Figure 6:**
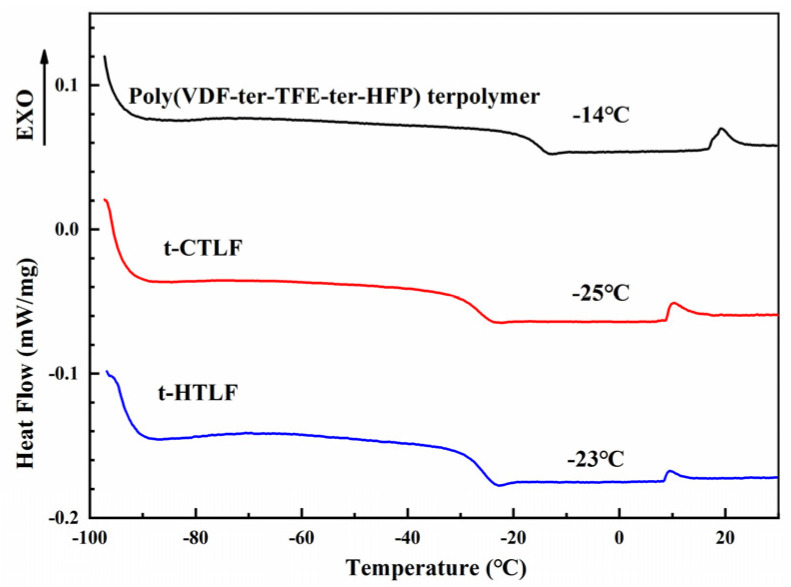
DSC of poly(VDF-*ter*-TFE-*ter*-HFP) terpolymer, t-CTLF and t-HTLF.

Based on the structural analysis, the thermal properties of the t-HTLF were tested and compared with those of the poly(VDF-*ter*-TFE-*ter*-HFP) terpolymer and t-CTLF; the results are shown in Figure 6. The T_g_ of both liquid end-group functionalised fluoroelastomers prepared from the poly(VDF-*ter*-TFE-*ter*-HFP) terpolymer are significantly lower than that of the raw material, which decreases from −14 °C to −25 °C and −23 °C, respectively. This is because, after the oxidative degradation reaction, the molar mass of t-CTLF is 3.8 × 10^3^ g/mol, lower than poly(VDF-*ter*-TFE-*ter*-HFP) terpolymer and the existence of isolated double bonds in the molecular chain. These factors will increase the flexibility of the molecular chain, so the T_g_ of t-CTLF decreases. After the reduction reaction, the C=C in the molecular chain are reduced to single bonds, which reduces the number of isolated C=C and flexibility of the molecular chain, thereby increasing the T_g_ of the t-HTLF [56]. Simultaneously, this maintains a good fluidity at low temperatures.

**Figure 7:**
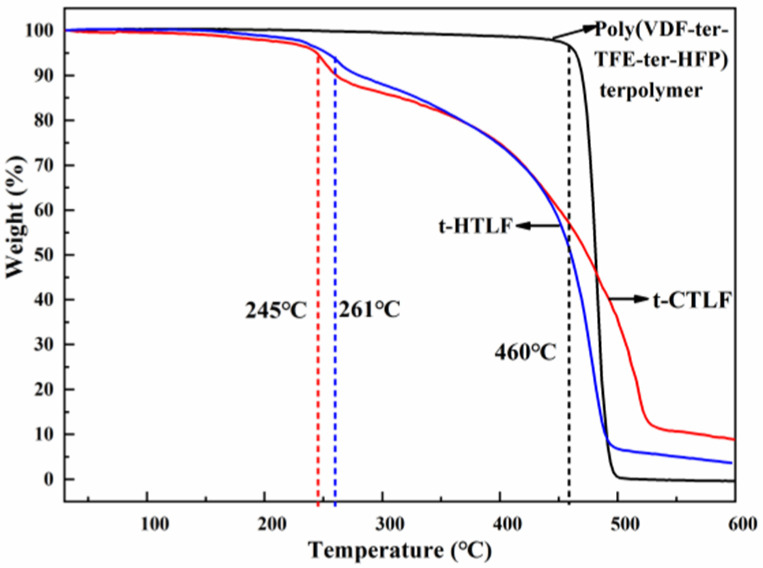
TGA of the poly(VDF-*ter*-TFE-*ter*-HFP) terpolymer, t-CTLF and t-HTLF.

The TGA results for the poly(VDF-*ter*-TFE-*ter*-HFP) terpolymer, t-CTLF, and t-HTLF are shown in Figure 7. The heat resistance of the two liquid fluoroelastomers prepared from the poly(VDF-*ter*-TFE-*ter*-HFP) terpolymer is significantly reduced, with T_d_ decreasing from 460 °C to 245 °C for the t-CTLF and 261 °C for the t-HTLF, respectively. This may also be attributed to the reduction of C=C to single bonds, which causes a significant decrease in T_d_. In addition to the molar mass and monomer composition of the liquid fluoroelastomers, C=C in the molecular chains also influences the thermal characteristics.

### 3.3. Factors Influencing the Reaction

Because LiAlH_4_ is slightly soluble in THF, the effects of the solvent dosage, reaction time, reaction temperature, and reductant dosage on the experimental results were systematically investigated.

#### 3.3.1. Solvent Dosage

The reaction condition was 80 °C × 4 h, the molar ratio of COOH/LiAlH_4_ was 1.00/4.00, and the amount of solvent was changed, No. 1, No. 2, No. 3, No. 4, No. 5. and No. 6 corresponded to THF dosage of 30 mL, 50 mL, 70 mL, 90 mL, 100 mL and 120 mL. The effect of solvent dosage on the product properties was studied, and the results were shown in Table 3. At a solvent dosage of 100 mL, the carboxyl conversion was high, reaching 94%. Therefore, the optimal solvent dosage was 100 mL of THF.

#### 3.3.2. Reaction Temperature

The effects of the reaction temperature on the attributes of the products were presented in Table 4, where the molar ratio of COOH/LiAlH_4_ was 1.00/4.00, the volume of the solvent was 100 mL, the reaction time was 4 h, and reaction temperature was changed, No. 7, No. 8, No. 9, No. 10 and No. 11. corresponded to reaction temperature of 70 °C, 80 °C, 90 °C, 100 °C and 120 °C. As shown in Table 4, the carboxyl conversion first increased with the reaction temperature before decreasing. At 80 °C, the carboxyl conversion was 94%. The t-HTLF colour gradually deepened and the molar mass increased with the reaction temperature, indicating the occurrence of side reactions. Therefore, 80 °C was chosen as the optimal reaction temperature.

#### 3.3.3. Reaction Time

The effects of reaction time on the characteristics of the products were examined at a reaction temperature of 80 °C, molar ratio of COOH/LiAlH_4_ of 1.00/4.00, and 100 mL of the solvent, and reaction time was changed, No.12, No.13, No.14, No.15 and No.16. corresponded to reaction time of 2 h, 4 h, 6 h, 8 h and 10 h. The results obtained under these conditions are listed in Table 5. The reduction efficiency increased with the reaction period, and the carboxyl conversion was the most significant at a reaction time of 4 h. The carboxyl conversion remained constant when the reaction time was increased to 6 h. Thus, 4 h was the ideal reaction time.

#### 3.3.4. Reductant Ratio

**Figure 8:**
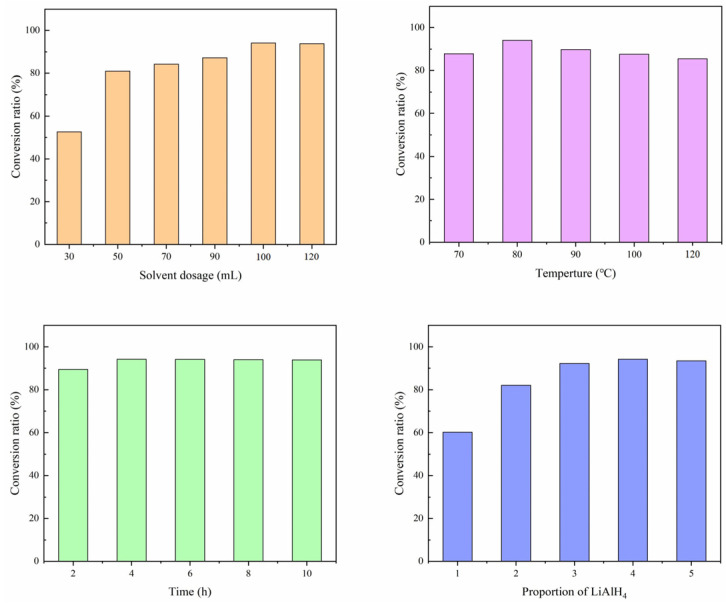
Effect of different factors on product conversion.

The influence of the COOH/LiAlH_4_ reduction system on the product properties was examined, and the results were presented in Table 6. The reaction conditions were 80 °C, 4 h, and 100 mL of solvent dosage, with other parameters remaining the same. Table 6 showed that the carboxyl content steadily decreased when the reductant dosage was increased. The carboxyl conversion was the maximum at 94% at a COOH/LiAlH_4_ molar ratio of 1.00/4.00, and it stabilized with a further increase in the reduction system dosage. The ideal COOH/LiAlH_4_ molar ratio for the reductant dosage was 1.00/4.00. In summary, Figure 8 shows the effect of different factors on product conversion.

### 3.4. Curing of the Hydroxyl-Terminated Liquid Fluoroelastomer

#### 3.4.1. Structural Characterisation

**Figure 9:**
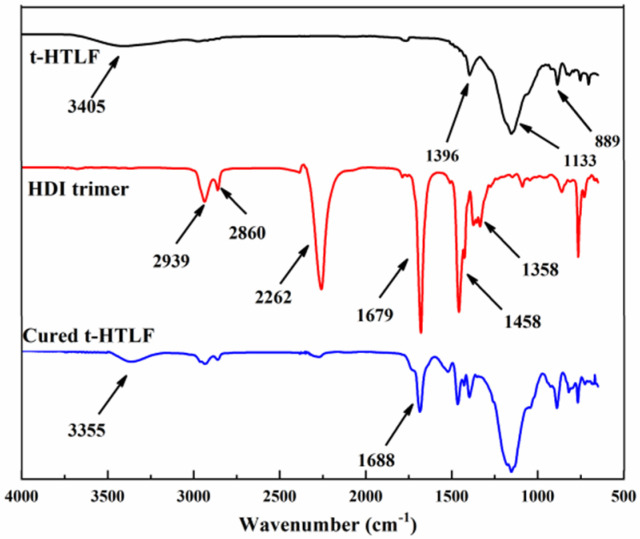
FTIR spectra of the t-HTLF, HDI trimer, and cured t-HTLF.

Structural characterisation of the t-HTLF, HDI trimers, and cured products was performed using FTIR, and the results are shown in Figure 9. In the FTIR spectra, the stretching vibration peaks corresponding to imino (-N-H-) and carbonyl group (C=O) in the carbamate (-NHCOO-) structure appear at 3355 and 1688 cm^−1^, respectively. This indicates that t-HTLF undergoes a curing cross-linking reaction with the HDI trimers.

**Figure 10:**
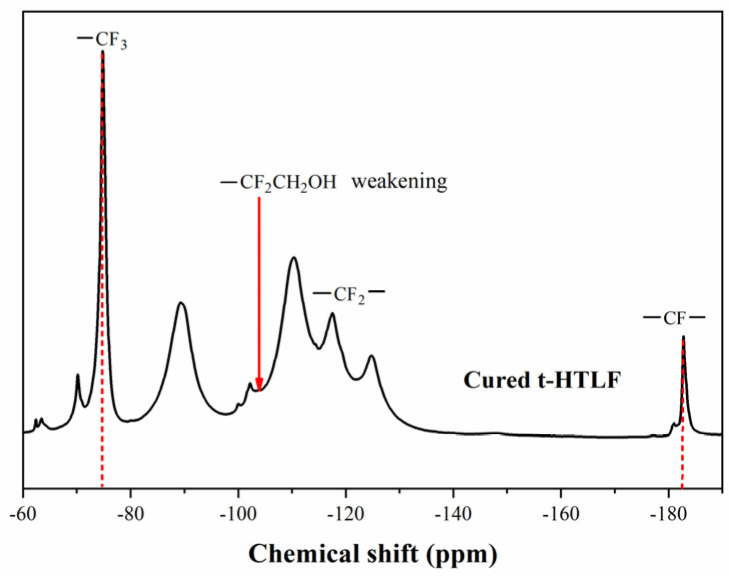
Solid-state ^19^F-NMR spectrum of the cured t-HTLF.

The ^19^F-NMR spectrum of the cured t-HTLF is shown in Figure 10. The peak centred at −75 ppm is assigned to -CF_3_ groups from the HFP sequences; the multiplets in the range of −83 to −165 ppm is attributed to the -CF_2_- groups from the HFP, VDF, and TFE sequences; and the peak centred at −185 ppm is assigned to the -CF- groups from the HFP sequences. Moreover, the characteristic peak corresponding to the -CF_2_CH_2_OH structure at −104.02 ppm is significantly weaker, indicating the reaction of the OH in the t-HTLF with the NCO in the HDI trimer to form -NHCOO-, concluding the cross-linking reaction and curing the liquid fluoroelastomer. These results are consistent with the FTIR results. The result was further confirmed by the solid-state ^19^F-NMR spectrum, and the characteristic peak area corresponding to the VDF sequence structure was ∑CF_2_ = I_−63.42_ + I_−89.28_ + I_−102.16_ + I_−110.34_ + I_−117.45_, the distinct peak area corresponding to the HFP sequence structure was ∑CF_3_ = I_−70.17_ + 2(I_−74.79_), and the distinct peak area corresponding to the TFE sequence structure was ∑CF_2_ = I_−124.80_. According to the Equations (5)–(8), it can be calculated that the contents of VDF, HFP and TFE in t-CTLF were 73%, 10%, 12%, respectively. The fluorine content in the cured t-HTLF was calculated to decrease after the curing process, from 67% to 65%.

#### 3.4.2. Curing Reaction Mechanism

**Figure 11:**
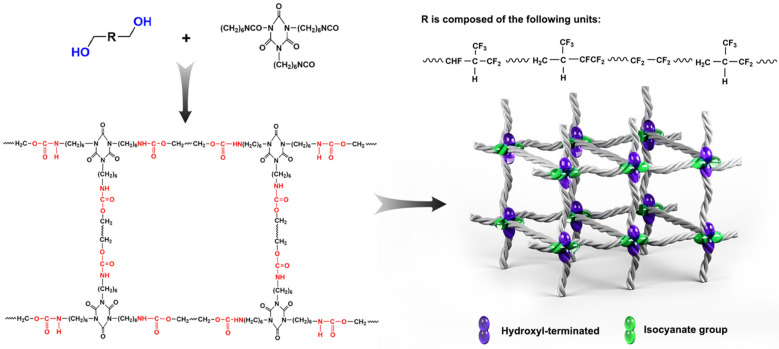
Curing reaction mechanism.

Because of the higher electron-cloud density and electronegativity of the N and O atoms in NCO, the C atom has a correspondingly higher positive charge. It is more vulnerable to the attack of a nucleophilic reagent. The process proceeds with further expansion of the molecular chain to form a mesh structure and complete the solidification reaction, as the active H atoms in the t-HTLF attack the C atoms of NCO to produce -NHCOO- via a nucleophilic addition reaction [36,57,58,59]. The curing reaction mechanism is shown in Figure 11.

#### 3.4.3. Mechanical Properties of the Cured Hydroxyl-Terminated Liquid Fluoroelastomer

By keeping the reaction conditions and amount of curing agent constant, the mechanical properties of the cured t-HTLF were investigated and compared with those of the cured c-HTLF under the same conditions. The results are shown in Table 7. The tensile strength of the cured t-HTLF is 2.15 MPa after curing, and the elongation at break is 200%, which is better than that of the cured c-HTLF [42]. This indicates that hydroxyl content and monomer composition affect the mechanical properties of cured products. Because t-HTLF contains TFE, its molecular chain exhibits higher polarity and rigidity; therefore, the tensile strength of its cured products is higher.

#### 3.4.4. Thermal Stability

**Figure 12:**
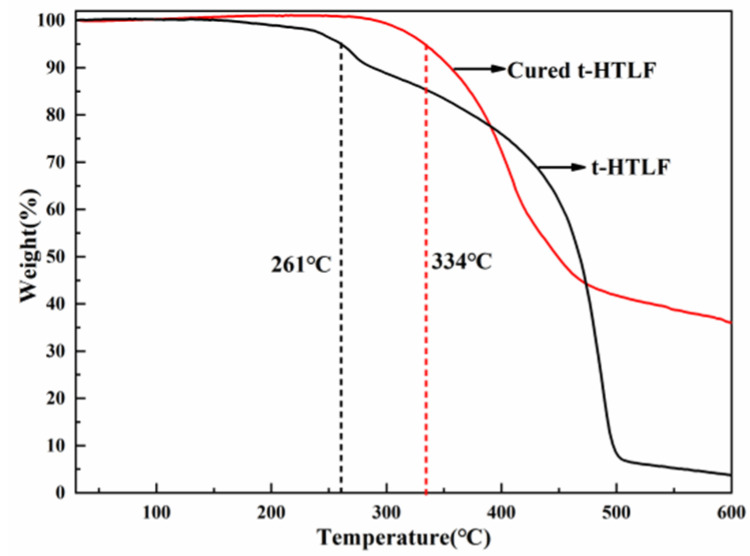
TGA thermograms of the t-HTLF and cured t-HTLF.

The thermal stability of the cured t-HTLF was tested using TGA for a t-HTLF and OH/NCO molar ratio of 1.00/1.20, and the results are shown in Figure 12. Before and after the curing of the t-HTLF, T_d_ increases from 261 °C to 334 °C, and the residual carbon content at 600 °C increased from 3.82% to 35.96%. This indicates that the presence of TFE in the t-HTLF further enhanced the thermal stability of the cured t-HTLF [60,61].

#### 3.4.5. Surface Properties

**Figure 13:**
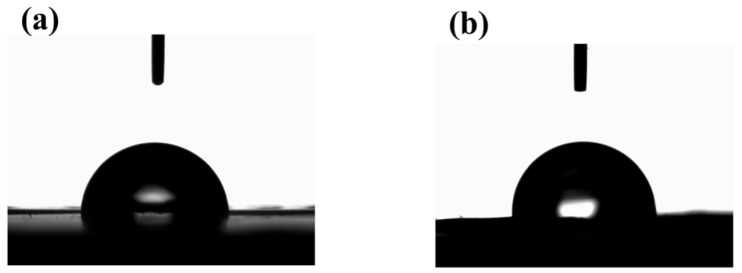
Water contact angle of the cured t-CTLF (**a**) and cured t-HTLF (**b**).

Since the OH activity in the t-HTLF is higher than that of COOH, its surface energy is lower and it exhibits hydrophobicity [62]; therefore, the contact angle of the cured t-HTLF is higher, reaching 90°. In comparison, the contact angle of the cured t-CTLF is 85°, cured t-HTLF exhibits stronger hydrophobicity than the cured t-CTLF. The specific results are shown in Figure 13.

#### 3.4.6. Oil and Acid Resistances

**Figure 14:**
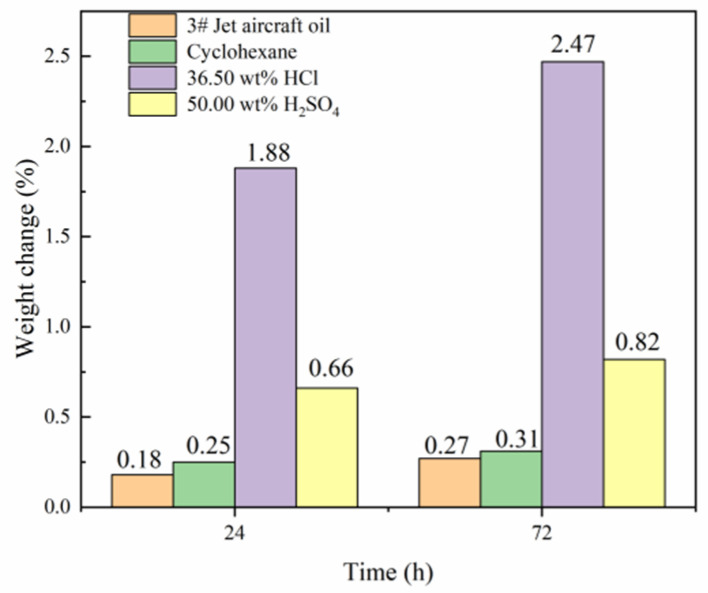
Change in the mass of the cured t-HTLF.

The oil and acid resistances of the cured t-HTLF were tested and compared with those of cured t-CTLF, and the results are shown in Figure 14. The rate of change of mass of the cured t-HTLF in these media after 24 and 72 h is also less than 5.00%, indicating that the cured t-HTLF had better oil and acid resistances.

## 4. Conclusions

In summary, the molar mass of a poly(VDF-*ter*-TFE-*ter*-HFP) terpolymer decreases from 93.0 × 10^3^ g/mol to 3.8 × 10^3^ g/mol after the oxidative degradation reaction. The t-CTLF can be prepared via the oxidative degradation method, and there is a small amount of C=C in the chains of t-CTLF that is not entirely degraded via oxidative. This affects the chemical and thermal stability of the t-CTLF and even causes side reactions in the subsequent functional reaction of the liquid fluoroelastomers. The efficient reduction system of LiAlH_4_ developed in this study can not only achieve an efficient conversion of COOH in the t-CTLF to OH but also the in situ hydrogenation addition reaction of the residual C=C in the chain. Thus, the thermal stability and end-group activity of the product are improved while maintaining a high fluorine content.

Furthermore, when the reaction conditions are 80 °C, 4 h, and a COOH/LiAlH_4_ molar ratio of 1.00/4.00, t-CTLF is reduced to t-HTLF via LiAlH_4_. The end-group conversion rate can reach 94%. The molar mass increases from 3.8 × 10^3^ g/mol to 4.2 × 10^3^ g/mol owing to the hydrogenation and addition reaction of C=C to the molecular chain. The T_g_ of t-HTLF is −23 °C, which is 2 °C higher than that of t-CTLF. Thus, it exhibits good fluidity at low temperatures. The T_d_ of t-HTLF is 261 °C, which exhibits a good thermal stability.

After curing, various end groups of the liquid fluoroelastomers exhibit distinct curing efficiencies and properties. Because the activity of OH is higher than that of COOH, OH can be cured efficiently in a shorter time. The T_d_ of cured t-HTLF can be as high as 334 °C, which is approximately 73 °C higher than that of the cured t-CTLF. In addition, its tensile strength is 2.15 MPa and elongation at break is 200%. Moreover, both fluoroelastomers exhibit hydrophobicity, and the WCA of the cured t-HTLF is 90°. The higher the activity of an end group, the stronger its hydrophobicity. The cured t-HTLF possesses high oil and acid resistances, and the fluorine content in the cured t-HTLF is well kept above 65%, and decreases from 67% to 65% after curing.

## Figures and Tables

**Table 1:** Assignments of ^19^F-NMR peaks in poly(VDF-*ter*-TFE-*ter*-HFP) terpolymer, t-CTLF and t-HTLF.

No.	δ (ppm)	Assignment	No.	δ (ppm)	Assignment
a	−63.53	–CF_2_CF_2_COOH	o	−113.85	–CF_2_CH_2_CF_2_CF_2_CH_2_–
b	−70.76	–CH_2_CF_2_CF(CF_3_)CF_2_CH_2_–	p	−117.26	–CH_2_CH_2_CF_2_CF_2_CH_2_–
c	−74.29	–CF_2_CH=C(CF_3_)CF_2_–	q	−118.68	–CF_2_CF_2_CF(CF_3_) CH_2_–
d	−75.44	–CF_2_CH_2_CF(CF_3_)CF_2_CF_2_–	r	−121.13	–CF_2_CF_2_CF_2_CF_2_CF_2_–
e	−80.53	–CH=CFCF(CF_3_)–	s	−122.90	–CH_2_CF_2_CF_2_CF_2_CF_2_–
f	−81.19	–CF=CHCF(CF_3_)CF_2_–	t	−123.58	
g	−91.95	–CF_2_CH_2_CF_2_CH_2_CF_2_–	u	−124.54	–CH_2_CF_2_CF_2_CF_2_CH_2_–
h	−93.28	–CF_2_CH_2_CF_2_CH_2_CF(CF_3_)–	v	−124.97	
i	−95.64	–CH_2_CH_2_CF_2_CH_2_CF_2_–	w	−125.19	
j	−103.48	–CF_2_CH_2_CF_2_CF(CF_3_)CF_2_–			
k	−104.02	–CF_2_CH_2_OH	x	−125.59	
l	−108.98	–CF(CF_3_)CH_2_CF_2_CF_2_CF(CF_3_)–			
m	−109.85	–CF_2_CH_2_CF_2_CF_2_CF(CF_3_)–	y	−126.18	
n	−111.64	–CF(CF_3_)CH_2_CF_2_CF_2_CH_2_–	z	−182.37	–CH_2_CF_2_CF(CF_3_)CF_2_CH_2_–
			a′	−184.32	–CF_2_CF_2_CF(CF_3_)CH_2_CF_2_–

**Table 2:** Assignments of GPC in liquid fluoroelastomers.

Sample	M_n_ × 10^3^ (g/mol)	PD
Poly(VDF-*ter*-TFE-*ter*-HFP) terpolymer	93.0	1.91
t-CTLF	3.8	1.65
t-HTLF	4.2	1.57

PS is used as the standard sample.

**Table 3:** Effect of THF on the product characteristics.

No.	THF (mL)	M_n_ × 10^3^ (g/mol)	PD	Conversion Ratio (%)
1	30	4.1	1.72	52
2	50	4.1	1.65	80
3	70	4.1	1.66	84
4	90	4.1	1.52	87
5	100	4.2	1.57	94
6	120	4.2	1.55	93

**Table 4:** Effect of reaction temperature on the product characteristics.

No.	Temperture (°C)	M_n_ × 10^3^ (g/mol)	PD	Conversion Ratio (%)
7	70	4.2	1.52	87
8	80	4.2	1.57	94
9	90	4.2	1.52	89
10	100	4.2	1.60	87
11	120	4.4	1.59	85

**Table 5:** Effect of reaction time on the product characteristics.

No.	Time (h)	M_n_ × 10^3^ (g/mol)	PD	Conversion Ratio (%)
12	2	3.9	1.68	89
13	4	4.2	1.57	94
14	6	4.3	1.61	94
15	8	4.3	1.60	94
16	10	4.3	1.60	93

**Table 6:** Effect of molar ratio of COOH/LiAlH_4_ on the product characteristics.

No.	COOH/LiAlH_4_	M_n_ × 10^3^ (g/mol)	PD	Conversion Ratio (%)
17	1.00/1.00	4.2	1.68	60
18	1.00/2.00	4.1	1.98	82
19	1.00/3.00	4.0	1.59	92
20	1.00/4.00	4.2	1.57	94
21	1.00/5.00	3.9	1.61	93

**Table 7:** The mechanical properties of different cured fluoroelastomers.

Liquid Fluoroelastomer	M_n_ × 10^3^ (g/mol)	OH (wt%)	Tensile Strength (MPa)	Elongation at Break (%)
Poly(VDF-*co*-HFP) copolymer	4.0	2.33	1.23	125
Poly(VDF-*ter*-TFE-*ter*-HFP) terpolymer	4.2	3.05	2.15	200

## Data Availability

Not applicable.

## References

[1] Ameduri B., Boutevin B., Kostov G. (2001). Fluoroelastomers: Synthesis, properties and applications. Prog. Polym. Sci..

[2] Zhao Y. (2013). Progress on research and application of special polymer materials in aerospace industry. Mater. Chin..

[3] Li D.H., Liao M.Y. (2018). Dehydrofluorination Mechanisms and Structures of Fluoroelastomersin Alkaline Environments. Mater. Rep..

[4] Zhang Z.Z. (2015). Preparation of Fluorinated Polyphosphazene Elastomer, Formulation Optimization and Properties Study. Master’s Thesis.

[5] Flitney B. (2005). Extending the application of fluorosilicone elastomers. Seal. Technol..

[6] Lee P.C., Kim S.Y., Ko Y.K., Ha J.U., Jeoung S.K., Lee J.Y., Kim M. (2022). Durability and Service Life Prediction of Fluorocarbon Elastomer under Thermal Environment. Polymers.

[7] Kang Q., Li A., Zhang X. (2021). Study on mechanical and tribological properties of cerium oxide/fluorine rubber composites. Mater. Today Commun..

[8] Calabrese L., Alenza A. (2003). The effect of a liquid rubber modifier on the thermo-kinetic parameters of an epoxy resin during a pultrusion process. Compos. Sci. Technol..

[9] Maclachlan J.D. (1978). Fluorocarbon elastomers: A technical review. Polym-Plast. Technol..

[10] Pianca M., Bonardelli P., Tatò M., Cirillo G., Moggi G. (1987). Composition and sequence distribution of vinylidene fluoride copolymer and terpolymer fluoroelastomers. Determination by ^19^F nuclear magnetic resonance spectroscopy and correlation with some properties. Polymer.

[11] Li J., Lv Y.F., You W.T., Guo L.L., Liu C., Zhang X.A., Qi S.C. (2013). Synthesis, cure and properties of siloxy-terminater liquid fluoroelastomers. Acta Polym. Sin..

[12] Souzy R., Boutevin B., Ameduri B. (2012). Synthesis and characterizations of novel proton-conducting fluoropolymer electrolyte membranes based on poly (vinylidenefl- uoride-ter-hexafluoropropylene-ter-α -trifluoromethacrylicacid) terpolymers grafted by arylsulfonic acids. Macromolecules.

[13] Chalathorn C., Kui X., He H.J. (2010). Proton-conductive polymer nanocomposite membranes prepared from telechelic fluorinated polymers containing perfluoro- sulfonic acid sidechains. Polym. Sci. Pol. Chem..

[14] Li K., Liang S., Lu Y. (2007). Synthesis of telechelic fluoroelastomers with well-defined functional end groups for cross-linked networks and nanocomposites. Macromolecles.

[15] Wang Y., Bai Y.P., Yue L.P., Zheng X.Q. (2019). Synthesis of photo-crosslinked hybrid fluoropolymer and its application as releasing coating for silicone pressure-sensitive adhesives. Appl. Polym. Sci..

[16] Guan F.X., Wang J., Yang L.Y. (2011). Confinement-induced high-field antiferroelectric-like behavior in a poly(vinylidene fluoride-co-trifluoroethylene-cochlorotrifluoroeth-ylene)-graft-polystyrene graft copolymer. Macromolecules.

[17] Li D.H., Liao M.Y. (2017). Study on the synthesis of liquid terminal acylfluorine rubber from different acylated reagents. Chin. Rubb/Plast. Technol. Equip..

[18] Li D.H., Qi S.C., Zhang X.A., Liao M.Y. (2016). Preparation, Functionalization and Properties of Low Molecular Fluoropolymers. Prog. Chem..

[19] Gallagher G.A. (1962). Copolymers of Hexalfuoropropylene Vinylidene Fluoride and Aliphatic, Chain Transfer Agents. US Patent.

[20] Ameduri B. (2001). From vinylidene fluoride (VDF) to the applications of VDF-containing polymers and copolymers: Recent developments and future trends. Chem. Rev..

[21] Tatemoto M., Morita S. (1982). Liquid Fluorine-Containing Polymer and its Production with Iodinated Compound. U.S. Patent.

[22] Hung M.H. (1993). Iodine Containing Chain Transfer Agents for Fluoropolymer Polymerizations. U.S. Patent.

[23] Kostov G.K., Holan M., Ameduri B., Hung M.H. (2012). Synthesis and Characterizations of Photo-Cross-Linkable Telechelic Diacrylate Poly(vinylidene fluoride-co-perfluoromethyl vinyl ether) Copolymers. Macromolecules..

[24] Li J., Lv Y.F., Qi S.C. (2014). Synthesis and cure properties of liquid fluoroelastomer with carboxyl end group. Chin. Synth. Rubb. Ind..

[25] Li D.H., Liao M.Y. (2018). Study on the dehydrofluorination of vinylidene fluoride (VDF) and hexafluoropropylene (HFP) copolymer. Polym. Degrad. Stabil..

[26] Li D.H., Liao M.Y. (2017). Dehydrofluorination mechanism, structure and thermal stability of pure fluoroelastomer (poly(VDF-ter-HFP-ter-TFE) terpolymer) in alkaline environment. J. Fluor. Chem..

[27] Mitra S., Ghanbari-Siahkali A., Kingshott P., Almdal K., Christensen A.G. (2003). Chemical degradation of fluoroelastomer in an alkaline environment. Polym. Degrad. Stabil..

[28] Liao M.Y., Qi R.R., Huo Y., Chang Y.F. (2022). Preparation and Characterization of Liquid Carboxyl-Terminated Fluororubberby Microwave Assisted Oxidation Degradation Method. Polym. Mater. Sci. Eng..

[29] Khalilzadeh M.A., Hosseini A., Tajbakhsh M., Mohannazadeh F. (2008). LiAlH_4_/silica chloride as a new chemoselective system for reduction of carbonyl compounds and phosphine oxides. J. Iran. Chem. Soc..

[30] Mariappan P., Muniappan T. (2000). Methods of enhancement of reactivity and selectivity of sodiumborohydride for applications in organic synthesis. J. Organomet. Chem..

[31] Tale R.H., Patil K.M., Dapurkar S.E. (2003). An extremely simple, convenient and mild one-pot reduction of carboxylic acids to alcohols using 3,4,5-trifluorophenylboronic acid and sodium borohydride. Tetrahedron. Lett..

[32] Saavedra J.Z., Resendez A., Rovira A., Eagon S., Haddenham D., Singaram B. (2012). Reaction of InCl_3_ with various reducing agents: InCl_3_-NaBH_4_-mediated reduction of aromatic and aliphatic nitriles to primary amines. J. Org. Chem..

[33] Aramini A., Brinchi L., Germani R., Savelli G. (2000). Reductions of α,β-Unsaturated Ketones by NaBH_4_ or NaBH_4_ + CoCl_2_: Selectivity Control by Water or by Aqueous Micellar Solutions. Eur. J. Org. Chem..

[34] Améduri B., Balagué J., Boutevin B., Caporiccio G., Gornowicz G. (1992). Synthesis and characterization of fluorinated telomers from vinylidene fluoride, hexafluoropropene and chlorotrifluoroethylene. J. Fluor. Chem..

[35] Wu J.J. (2015). Synthesis and Cure of Active-Terminatedliquid Flouroelastomer. Master’s Thesis.

[36] Li N., Wu J.J., Wang Y.Q., Wu J.H., Zhang J.J., Huo J.Z., Bai Y.P. (2017). Synthesis, curing of hydroxyl-terminated liquid fluoroelastomer: Thermal, chemical resistant and mechanical properties. IOP. Conf. Ser. Mater. Sci. Eng..

[37] Li D.H. (2018). Preparation, Curing, Structures and Properties of End-functionalized Liquid Fluoroelastomers. Ph.D. Thesis.

[38] Chang Y.F., Liao M.Y., Li X.Y. (2020). Reduction of liquid terminated-carboxyl fluoroelastomers using NaBH_4_/SmCl_3_. RSC Adv..

[39] Chang Y.F., Liao M.Y., Wen J.M. (2022). Reduction Performance and Mechanism of Liquid Terminated-carboxyl Fluoroelastomers Using NaBH_4_/MCl_x_ Reduction System. Chem. J. Chin. Univ.-Chin..

[40] Song A., Zhang X., Song X., Chen X., Yu C., He H., Li H., Wei W. (2014). Construction of Chiral Bridged Tricyclic Benzopyrans: Enantioselective Catalytic Diels-Alder Reaction and a One-Pot Reduction/Acid-Catalyzed Stereoselective Cyclization. Angew. Chem..

[41] Schwager H., Wilke G. (2010). Zum Norcaradien-Cycloheptatrien-Strukturproblem. Eur. J. Inorg. Chem..

[42] Duan J.Y., Yang C., Kang H.L., Li L., Yang F., Fang Q.H., Han W.C., Li D.H. (2022). Structure, preparation and properties of liquid fluoroelastomers with different end groups. RSC Adv..

[43] Wen J.M., Chang Y.F., Liao M.Y. (2022). Reduction of Liquid Carboxyl-terminated Fluorine Rubber by Alkyl Aluminum/Lithium Aluminum Hydride Reduction System. Chin. Rubb. Ind..

[44] Li A.J., Feng B., Liu Q.C. (2011). Synthesis of 1,3-Aminoalcohols by One-Step Reduction of β-Enaminoketoneswith Lithium Aluminium Hydride. Chin. J. Org. Chem..

[45] Zhai H. (2007). Synthesis of Antibacterials Moxifloxacin. Master’s Thesis.

[46] Herold F., Leubner O., Pfeifer P., Zakgeym D., Drochner A., Qi W., Etzold B.J. (2021). Synthesis strategies towards amorphous porous carbons with selective oxygen functionalization for the application as reference material. Carbon.

[47] Wang Y., Liu H., Zheng X., Bai Y. (2019). Towards new environmentally friendly fluoroelastomers: From facile chemical degradation to efficient photo-crosslinkable reaction. Polym. Int..

[48] Timperley C.M., Arbon R.E., Bird M., Brewer S.A., Willis C.R. (2003). Bis (fluoroalkyl) acrylic and methacrylic phosphate monomers, their polymersand same of their properties. J. Fluor. Chem..

[49] Saint L.R., Manseri A., Ameduri B., Lebret B., Vignane P. (2002). Synthesis and properties of novel fluorotelechelic macrodiols containing vinylidene fluoride, hexafluoropropene and chlorotrifluoroethylene. Macromolecules.

[50] Li D.H., Liao M.Y. (2017). Preparation of telechelic hydroxyl low molecular weight fluoropolymers. Key Eng. Mater..

[51] Pianca M., Barchiesi E., Esposto G., Radice S. (1999). End groups in fluoropolymers. J. Fluor. Chem..

[52] Li D.H., Liao M.Y. (2018). Monomers Composition, Molecular Chain Structures and Thermal Propertiesof PLV85540 Fluoroelastomer. Mater. Rep..

[53] Xing Q.Y., Pei W.W., Xu R.Q., Pei J. (2017). Basic Organic Chemistry.

[54] Lin S.B., Li Z.Y., Luo G., Chen Y.Y. (2014). Methods of reducing carboxylic acid ester to alcohol. Chem. Ind. Eng. Prog..

[55] Guo J.Z., Huang P., Yang T., Pei W. (2015). Production and Reaction of Lithium Aluminium Hydride. Chem. Prod. Technol..

[56] Hua Y.Q., Jin R.G. (2013). Polymer Physics.

[57] Zeng N., Xie J.J., Ding C., Liu J.X. (2014). Synthesis and properties of blocked isocyanate adducts from methylparaben. Chem. Ind. Eng. Prog..

[58] Savoie H.R., Peticolas W.L. (2010). Curing reactions in elastomers. II. Carboxy-terminated polybutadiene. J. Appl. Polym. Sci..

[59] Sun H., Ni W., Yuan B., Wang T., Li P., Liu Y., Wang L. (2013). Synthesis and characterization of emulsion-type curing agent of water-borne epoxy resin. J. Appl. Polym. Sci..

[60] Li D.H., Duan J.Y., Zhang C., Xv Z.Y., Fang Q.H. (2021). Multi-stage Curing Mechanisms, Structure and Properties of 26/246Fluoroelastomers. Mater. Rep..

[61] Li X.Y., Chang Y.F., Liao M.Y. (2020). Study on Properties of Carboxyl-terminated Liquid Fluororubber Cured by Aziridine. Mater. Rep..

[62] Li H., Yao M., Lai J., Ma C.P., Wu Z.D., Qiu Z.M. (2023). Synthesis and Application of Hydroxy Terminated Fluorinated Vinyl Polysiloxane. Mater. Rep..

